# The effect of brief case management on emergency department use of frequent users in mental health: Findings of a randomized controlled trial

**DOI:** 10.1371/journal.pone.0182157

**Published:** 2017-08-03

**Authors:** Vicky Stergiopoulos, Agnes Gozdzik, Ashley Cohen, Tim Guimond, Stephen W. Hwang, Paul Kurdyak, Molyn Leszcz, Donald Wasylenki

**Affiliations:** 1 Centre for Addiction and Mental Health, Toronto, Canada; 2 Department of Psychiatry, University of Toronto, Toronto, Canada; 3 Centre for Urban Health Solutions, Li Ka Shing Knowledge Institute, St. Michael’s Hospital, Toronto, Canada; 4 Applied Health Research Centre (AHRC), The HUB, Li Ka Shing Knowledge Institute, St Michael's Hospital, Toronto, Canada; 5 Mental Health and Addictions Service, St. Michael’s Hospital, Toronto, Canada; 6 Division of General Internal Medicine, Department of Medicine, University of Toronto, Toronto, Canada; 7 Mount Sinai Hospital, Toronto, Canada; TNO, NETHERLANDS

## Abstract

Frequent users of hospital emergency departments (EDs) are a medically and socially vulnerable population. The Coordinated Access to Care from Hospital EDs (CATCH-ED) study examined the effectiveness of a brief case management intervention in reducing ED use and improving health outcomes among frequent ED users with mental health or addiction challenges in a large urban centre. Adults (≥18 years of age) who had five or more ED visits in the past 12-months, with at least one visit for mental health or addictions problems were randomized to either brief case management (N = 83) or usual care (N = 83) and followed for 12 months. The primary outcome of effectiveness was the frequency of ED visits during 12 months after study enrolment. Secondary outcomes included days in hospital, mental health and addiction symptom severity and health-related quality of life, measured by the SF-12. Compared to usual care, CATCH-ED participants saw a 14% reduction in frequency of ED visits during the 12-month post-randomization period [rate ratio (RR) = 0.86, 95% CI 0.64–1.15)], however, this finding did not reach statistical significance. There were also no statistically significant differences between the groups at 12 months in the number of days spent in hospital (RR = 1.16, 95% CI 0.59–2.29), physical (1.50, 95% CI -2.15–5.15) or mental (-3.97, 95% CI -8.13–0.19) component scores of the SF-12, severity of psychiatric symptoms (-0.41, 95% CI -2.30–1.49), alcohol (0.053 95% -0.017–0.12) or drug (-0.0027, 95% CI -0.0028–0.023) use. Compared to usual care, a brief case management intervention did not result in significantly reduced ED use or improved health outcomes among frequent ED users with mental health or addictions challenges in a large urban centre in Canada. Future studies need to evaluate the availability and accessibility of community-based resources for individuals with frequent ED use.

## Introduction

Frequent users of emergency departments (EDs), often defined as those with four or more visits in a year [1], comprise a small proportion of ED users (4.5–8%), but account for 21–28% of all ED visits [2]. Although frequent ED users represent a heterogeneous population with diverse health needs [2], studies across jurisdictions have noted several commonalities, including high rates of acute and chronic medical conditions, mental illness and addictions [3, 4], homelessness [5, 6] and perceived unmet mental health needs [7, 8], when compared to non-frequent ED users. According to a systematic review, presenting complaints are likewise varied among frequent ED users and include worsening of existing chronic conditions, pain, substance or mental health related challenges and a variety of different complaints upon each visit [2]. However, mental health or substance misuse related reasons are very common [9, 10], particularly among the most frequent ED users [11]. While several studies have shown that frequent ED use is mostly episodic in nature, with only approximately 25–30% of frequent users in a given year maintaining similar levels of ED use in the following year [12, 13], a recent study observed that among the group of most frequent ED users (those with 18 or more ED visits/year) in the province of Manitoba, 70% had the same level of ED use in the calendar year prior to the study [10]. Interestingly, this most frequent ED user group had the highest rate of a physician-based diagnosis of a mental illness, compared to less frequent users [10], suggesting that the highest frequency ED users comprise a population with high rates of mental illness, addiction and complex unmet needs.

Many jurisdictions have implemented interventions to reduce frequent ED use in effort to reduce costs and improve health outcomes for this population. To date, case management has been the most studied intervention for frequent ED users [1, 14], resulting in significant reductions in ED use in two prior randomized controlled trials (RCTs) [15, 16]. However, a recent meta-analysis noted that care coordination strategies, including case management, were only effective in reducing ED visits among older patients and were not effective in reducing health service use among patients with mental illness [17]. As mental illness contributes to frequent ED use across jurisdictions [18], there is an urgent need for interventions that address the unique needs of this population.

The goal of the CATCH-ED trial was to assess the effectiveness of brief intensive case management in reducing ED utilization among frequent ED users with mental health or addiction challenges, in Toronto, Canada, over 12 months of follow up. Secondary outcomes of interest included days in hospital, physical and mental health status, alcohol and drug use. Exploratory outcomes included disease-specific quality of life and the number of hospital admissions. We hypothesized that brief intensive case management would lead to reduced acute health care use and improved health outcomes in this population, when compared to usual care.

## Methods

### Study design

We conducted a non-blinded parallel-group randomised controlled trial of a brief intensive case management intervention for frequent ED users implemented across 6 EDs in Toronto, Canada. Participants were recruited from November 2012 and September 2013 and followed for 12 months. The study protocol (S1 File) was approved by the research ethics boards of all 6 participating hospitals, which are affiliated with the University of Toronto and the Toronto Academic Health Science Network: Centre for Addiction and Mental Health, St. Michael’s Hospital, St. Joseph’s Health Centre, Sunnybrook Health Sciences Centre, Toronto East General Hospital and University Health Network. The study was registered with ClinicalTrials.gov (NCT01622244) on June 4, 2012. The study design has been described in detail elsewhere [19].

### Setting

Toronto is Canada’s largest city, with 2.6 million residents in 2011 [20]], and has the highest concentration of health services in Canada [21]. Access to ED services is free for permanent residents of the province of Ontario and covered by the Ontario Health Insurance Plan (OHIP); with some minor exceptions (e.g. ambulance transports other than between hospitals). During 2013/14, the Toronto Central Local Health Integration Network (LHIN) reported that approximately 4.1% (N = 42,549) of all ED visits in central Toronto were for mental health and substance abuse-related reasons [21]. Based on 2013/14 data, repeat unscheduled ED visits for mental health and substance use reasons comprised 27.9% and 41.7%, respectively, of all repeat visits within 30 days [21].

### Participants

Study participants were identified by ED clinicians at participating hospitals either via frequent user lists or automated flagging systems, and referred to the study team with participant consent. Study eligibility was further assessed by a member of the research team, who obtained written informed consent from eligible participants prior to study enrolment. Study eligibility criteria included: adult age (≥ 18 years of age), a history of 5 or more ED visits in the past year to any one of 6-participating hospital EDs and at least one ED visits in the past year for a mental health or addictions-related condition. Participants who had a history of 5 or more ED visits in the past but did not have at least one visits for a mental health or addictions-related condition were excluded. All study participants provided written informed consent.

### Randomization

Randomization was performed upon completion of the eligibility screening and baseline interviews using a computer connected to the study data coordination centre. The participants’ allocation was communicated to participants as it was revealed, and those randomized to the intervention group were immediately referred to a CATCH-ED case manager. Block randomization was utilized, with a 1:1 allocation ratio and randomly selected block sizes, to maintain balance in the allocation of participants to the treatment groups at intermediate points in the recruitment process [22]. Although the study was not blinded due to the nature of the intervention, the primary outcome of ED utilization and other health care use outcomes were derived from administrative data held at the Institute for Clinical Evaluative Sciences (ICES).

### Procedures

Participants who met study inclusion criteria were randomly assigned to the CATCH-ED intervention or a usual care group and met with a member of the research team to complete a series of questionnaires at 3-month intervals over 12 months. Participant responses were uploaded to the data coordinating centre’s secure server, using tablet computers. Participant interviews were conducted in various locations, based on participant preference, including the research team’s office, service provider locations and public settings.

Participant interviews alternated between longer interviews at baseline, 6- and 12- months (approximately one hour) and shorter interviews at 3- and 9-months (approximately 15 min). In months without a scheduled interview, participants were contacted for a very brief monthly check-in. Participants were compensated with a cash honorarium ($25–50) and transit fare upon completion of each follow-up interview. To improve retention, participants were asked to provide current contact information for family members, friends and service providers, at each contact point.

### Intervention

Participants randomized to the intervention group were connected to CATCH-ED case managers, seconded from three community mental health agencies in Toronto. Case managers had access to a range of dedicated community support options, including primary care, peer support, mental health and addictions counselling, and other health and social services as needed, through partnerships with four community health centres and one peer outreach agency. A CATCH-ED program manager supervised case managers and ensured they maintained small caseloads of approximately 15 clients each. The CATCH-ED intervention was informed by the Critical Time Intervention (CTI) model [23, 24], an empirically supported time-limited case management intervention developed to prevent homelessness and other adverse outcomes for people with mental illness at the point of discharge from various institutions, including hospitals, shelters and prisons. CATCH-ED case managers worked with participants over 4–6 months to first foster engagement and identify needs and goals, secondly to connect participants to needed community-based services, and finally to transition and transfer participant care to longer-term community services [23, 24]. As part of the program, case managers offered outreach and home visits, crisis intervention, supportive therapy, practical needs assistance and care coordination, aiming to integrate hospital, community and social care and improve continuity of care.

### Usual care

All participants, including usual care participants, received an education session and a resource guide that described available community-based services and resources, which they were able to access at their discretion.

### Measures

The primary outcome measure was the frequency of ED visits during the 12-month period following randomization. Secondary outcomes assessed at 12 months included past month mental health symptom severity, measured by the modified Colorado Symptom Index [25]; alcohol and drug addiction severity, measured by the Addiction Severity Index (ASI) [26]; health-related quality of life [physical and mental component scores of the SF-12 [27] and the overall health EQ-5D Visual Analogue Scale (VAS) [28]] and length of stay (days) in hospital during the 12-month period following treatment allocation. Exploratory outcomes included disease-specific quality of life, measured using the QoLI-20 [29], both total summary score and a single global item at 12 months after randomization and the frequency of hospital admissions during the 12-month period following randomization.

All health care use outcomes (ED visits, length of hospital stay and hospital admissions) were analysed for consenting participants using administrative data held at the Institute of Clinical Evaluative Sciences (ICES). All other participant outcomes used participant self-report data captured during interviews with research staff.

More detailed descriptions of the measures are found elsewhere [19].

### Sample size

To account for the over-dispersed distribution of our primary outcome, as well as the natural reduction in ED visits over time, sample size was calculated using the usual care group outcomes of a previously published randomized controlled trial of case management among frequent ED users (≥5 visits/year) [15]. Using previously published formulas for recurring count outcomes [30], a 20% rate reduction would be detectable if each arm had 64 participants. To allow for up to 30% attrition, the final sample size was set at 83 participants in each arm, for a total of 166 participants. Sample size estimates were verified via simulation.

### Statistical methods

Data was analysed using the intent-to-treat principle. Of the 166 participants enrolled in the study, 160 (96%) gave consent to access their administrative health data records, of whom 159 were successfully linked (79 and 80, from the CATCH-ED and usual care arms, respectively) using personal health identifiers (unique OHIP numbers or name and date of birth). Count outcomes included administrative health care use data (ED visits, days in hospital, hospital admissions, number of primary care provider visits) which were modelled using an analysis of covariance framework that compared the number of outcome events in the CATCH-ED group compared to the TAU group during the 12-month post-randomization period, adjusting for baseline number of events accrued in the 12-month pre-randomization period, as well as participant age and sex. Models used a negative binomial distribution due to the over-dispersion of the data (variance greater than the mean) and generated rate ratios (RR) and their 95% CI for the 12-month post-randomization period. Rate ratios calculated the ratio of the estimated frequency of CATCH-ED events divided by the frequency of TAU events for the 12-month post-randomization period, adjusting for baseline frequencies.

Normally distributed outcomes included self-reported continuous secondary and exploratory outcomes which approximated the normal distribution (SF-12 physical and mental component summary scores, EQ-5D utility score, CSI summary score, QoLI-20 total score, Global item of QoLI20, ASI drug and alcohol composite scores) were analysed using an analysis of covariance framework to model difference in means (95% CI) between CATCH-ED and TAU groups at 12-month post-randomization, adjusting for baseline values.

All analyses were completed using R (https://www.r-project.org/). Significance level for all tests was set at 5%.

## Results

### Participants

**Fig 1:**
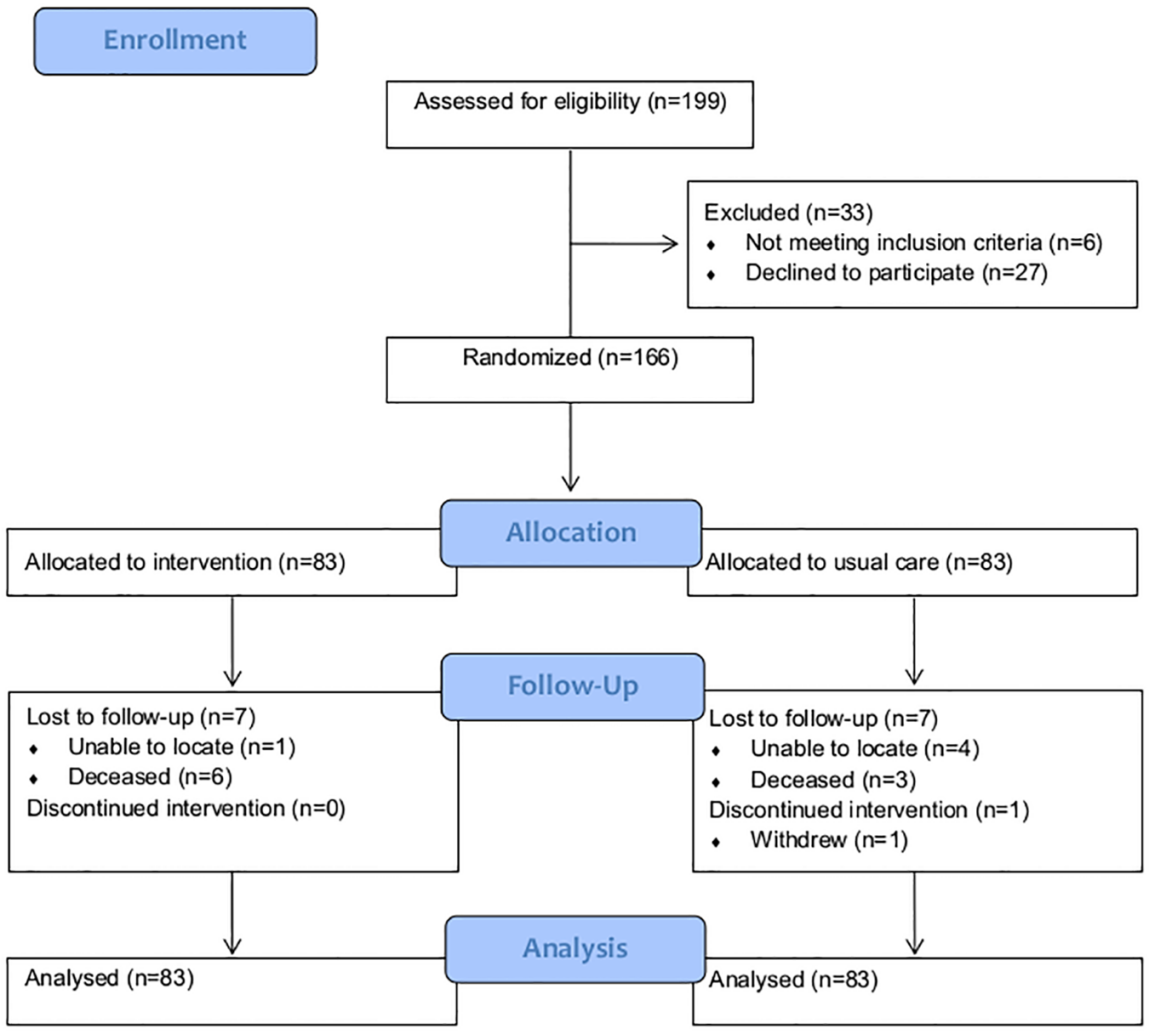
Participant flow through the study.

Between November 2012 and September 2013, the research team received a total of 271 unique referrals from participating hospital EDs. In total, 199 individuals were assessed for eligibility, 27 of whom declined to participate and 6 did not meet the inclusion criteria, resulting in a final enrolment sample of 166 participants (Fig 1). At 12 months’ post-randomization, the rate for completion of follow-up interviews was 91.6% (N = 76) and 90.4% (N = 75) for the usual care and CATCH-ED groups, respectively. The primary reasons for loss-to-follow-up were death (N = 6 for CATCH-ED and N = 3 for TAU participants) and inability to contact or locate the participant (N = 1 for CATCH-ED and N = 4 for TAU participants). Qualitative data collection was completed between August 2013 and December 2013 and reported separately [7]. The baseline characteristics of participants are shown in Table 1.

**Table 1:** Baseline characteristics of participants, stratified by randomization group.

		No. (%) or mean ± SD^1^	
Characteristics		CATCH-ED (n = 83)	Usual care (n = 83)
Age, years^2^		42.7 ± 15.7	47.1 ± 13.5
Male		39 (47.0%)	46 (55.4%)
Canadian-born		65 (78.3%)	58 (69.9%)
English primary language spoken		72 (86.8%)	73 (88.0%)
Racial or Ethnic Identity			
	Aboriginal	8 (10.0%)	6 (7.4%)
	Black	5 (6.3%)	8 (9.9%)
	Caucasian	52 (65.0%)	56 (69.1%)
	Other	15 (18.8%)	11 (13.6%)
Single, Never Married		58 (69.9%)	48 (57.8%)
High school or higher education		54 (65.9%)	51 (61.5%)
Unemployed		72 (87.8%)	76 (95.0%)
Receives disability income		62 (74.70%)	63 (75.9%)
Total income in past month, $CAD^3^		1227 ± 1003	1050 ± 569
Housed in the past 12 months^4^		63 (76.8%)	71 (86.6%)
Self-reported psychiatric disorders			
	Anxiety disorders^5^	48 (61.5%)	43 (55.8%)
	Mood disorders^6^	53 (63.9%)	47 (57.3%)
	Psychotic disorders^7^	21 (25.6%)	26 (32.5%)
	Substance misuse disorder^8^	44 (53.0%)	43 (53.8%)
	Personality disorder	20 (25.0%)	21 (25.9%)
3 or more self-reported comorbid conditions^9^		53 (63.9%)	56 (67.5%)
Has a regular medical doctor		68 (81.9%)	62 (74.7%)
Has a place to go when sick		76 (91.6%)	74 (89.2%)

Note: SD = standard deviation

^1^The following characteristics had missing values: education (n = 3), ethnicity (n = 5), employment (n = 4), income (n = 19) housing history (n = 2), has regular doctor (n = 1)

^2^ The median and interquartile range for age is 39.4 (28.4,54.0) and 48.1 (34.9,57.1) for CATCH-ED and for usual care, respectively.

^3^ The median and interquartile range for total income is 1025 (694, 1282) and 1065 (770,1200) for CATCH-ED and for usual care, respectively

^4^ Housed included residing in a group/boarding home, rooming house, on your own in apartment house, with other family members, with others, with spouse

^5^ Anxiety disorders include post-traumatic stress disorder

^6^ Major depressive disorder or bipolar affective disorder

^7^ Schizoaffective disorder or schizophrenia

^8^ Substance or alcohol misuse or dependence

^9^ Based on the Canadian Community Health Survey and the National Population Health Survey (http://www.statcan.gc.ca/)

### Primary outcomes

Compared to TAU, CATCH-ED participants had a 14% reduction in ED visits during the 12-month post-randomization period [rate ratio (RR) = 0.86, 95% CI 0.64–1.15)], adjusting for baseline values, age and sex. This reduction did not reach statistical significance (P = 0.31) (Tables 2 and 3).

**Table 2:** Study outcomes at baseline and 12 months, intent-to-treat analysis.

	Baseline				12 months			
	CATCH-ED (n = 83)		Usual care (n = 83)		CATCH-ED (n = 76)		Usual care (n = 75)	
	Mean SD	Median (IQR)	Mean SD	Median (IQR)	Mean SD	Median (IQR)	Mean SD	Median (IQR)
**Count outcomes**								
*Primary outcome*								
Emergency department (ED) visits	32.8 ± 45.9	19 (0,32)	27.2 ± 28.3	16 (10,35)	27.0 ± 41.9	12 (4,38)	25.7 ± 28.4	12 (5,38)
*Secondary outcomes*								
Days in hospital	5.8 ± 13.1	0 (0,4)	8.9 ± 19.4	0 (0,9)	6.2 ± 11.9	0 (0,8)	13.1 ± 32.1	1 (0,11)
*Exploratory outcomes*								
Hospital admissions	1.9 ± 4.1	0 (0,2)	2.4 ± 5.9	0 (0,3)	1.6 ± 3.7	0 (0,2)	2.4 ± 4.3	1 (0,3)
Number of primary care provider visits	12.5 ± 13.3	9 (2,17)	13.2 ± 13.8	10 (4,17)	9.8 ± 10.9	7 (2,12)	13.4 ± 15.0	9 (3,17)
**Normally distributed outcomes**								
*Secondary outcomes*								
Physical Component Score (SF-12)	40.1 ± 12.8	39.8 (29.6,49.4)	41.9 ± 12.0	43.0 (32.7,49.3)	42.8 ± 13.5	44.1 (31.1,53.0)	42.6 ± 11.6	43.0 (37.6,50.6)
Mental Component Score (SF-12)	33.5 ± 13.9	33.0 (22.5,41.4)	35.8 ± 13.0	33.2 (26.6,43.8)	38.3 ± 13.6	37.2 (29.7,49.2)	42.7 ± 13.8	43.8 (31.8,53.1)
Severity of mental health problems (CSI total score)	22.8 ± 6.0	25.0 (19.5,27.0)	21.4 ± 6.0	21 (18,26)	18.0 ± 6.33	18.0 (14.0,23.0)	17.1 ± 6.5	17 (12,21)
Alcohol composite score (ASI)	0.33 ± 0.33	0.23 (0.01,0.67)	0.25 ± 0.31	0.07 (0.00 to 0.52)	0.21 ± 0.28	0.06 (0.00,0.41)	0.15 ± 0.25	0.005 (0.00,0.03)
Drug composite score (ASI)	0.05 ± 0.09	0.00 (0.00,0.05)	0.07 ± 0.12	0.00 (0.00,0.10)	0.03 ± 0.06	0.00 (0.00,0.03)	0.04 ± 0.09	0.00 (0.00,0.03)
*Exploratory outcomes*								
Disease-specific quality of life (QoLI-20 total score)	79.5 ± 23.6	81.5 (62.5, 93.0)	89.0 ± 20.0	89 (76,99)	84.8 ± 19.9	87 (76,95)	96.1 ± 19.9	99 (82,111)
Global item (QoLI-20)	3.7 ± 2.1	4 (2,5)	4.2 ± 2.2	4 (3,6)	4.5 ± 1.7	5 (4,6)	4.4 ± 1.8	5 (3,6)
Overall health visual analogue scale (VAS) of the EQ-5D	54.2 ± 26.7	60.0 (32.5,75.0)	51.9 ± 26.7	50 (30,75)	59.1 ± 25.8	60.0 (50.0,75.2)	64.6 ± 25.3	70 (50,85)

**Table 3:** Differences (95% CI) between treatment arms (CATCH-ED—Usual care) at 12 months, intent-to-treat analysis.

**Count outcomes**^1^	**Rate ratio (95% CI)**	**p-value**
*Primary outcome*		
Emergency department (ED) visits	0.86 (0.64 to 1.15)	0.31
*Secondary outcomes*		
Days in hospital	1.16 (0.59 to 2.29)	0.66
*Exploratory outcomes*		
Hospital admissions	0.78 (0.46 to 1.30)	0.34
Number of primary care provider visits	0.83 (0.63 to 1.09)	0.18
**Normally distributed outcomes**^2^	**Mean difference (95% CI)**	**p-value**
*Secondary outcomes*		
Physical Component Score (SF-12)	1.50 (-2.15 to 5.15)	0.42
Mental Component Score (SF-12)	-3.97 (-8.13 to 0.19)	0.06
Severity of mental health problems (CSI total score)	-0.41 (-2.30 to 1.49)	0.68
Alcohol composite score (ASI)	0.053 (-0.017 to 0.12)	0.14
Drug composite score (ASI)	-0.0027 (-0.028 to 0.023)	0.84
*Exploratory outcomes*		
Disease-specific quality of life (QoLI-20 total score)	-9.12 (-17.57 to -0.67)	0.04
Global item (QoLI-20)	0.19 (-0.36 to 0.74)	0.51
Overall health visual analogue scale (VAS) of the EQ-5D	-4.17 (-12.4 to 4.03)	0.32

^1^ Count outcomes were modelled using an analysis of covariance framework, using a negative binomial distribution that compared the number of outcome events in the CATCH-ED group compared to the TAU group during the 12-month post-randomization period, adjusting for baseline number of events accrued in the 12-month pre-randomization period, as well as participant age and sex. Resulting rate ratios (RR) and their 95% CI calculate the ratio of the estimated frequency of CATCH-ED events divided by the frequency of TAU events for the 12-month post-randomization period, adjusting for baseline frequencies, age and sex.

^2^ Self-reported health outcomes which approximated the normal distribution were analysed using an analysis of covariance framework to model difference in means (95% CI) between CATCH-ED and TAU groups at 12-month post-randomization, adjusting for baseline values.

### Secondary outcomes

Similarly, adjusting for baseline values, the number of days spent in hospital (length of stay, LOS) during the 12-month post-randomization period did not differ statistically between the intervention and TAU group (RR = 1.16 95% CI 0.59–2.29).

There were no statistically significant differences between the groups at 12 months for SF-12 physical (1.50, 95% CI -2.15–5.15) or mental (-3.97, 95% CI -8.13–0.19) component scores, the overall health VAS of the EQ-5D (-4.17, 95% CI -12.4–4.03) and the severity of psychiatric symptoms (-0.41, 95% CI -2.30–1.49), adjusted for their corresponding baseline values. Similarly, there was no significant reduction in the severity of alcohol use or drug use, either when including all participants (0.053, 95% -0.017–0.12 and -0.0027, 95% CI-0.028–0.023, respectively) or when limiting analyses only to participants with ASI scores greater than zero on alcohol and drug use scales (0.061, 95% -0.039 to 0.051 and -0.0017, 95% CI -0.048 to 0.023, respectively), adjusting for baseline values.

### Exploratory outcomes

Neither the frequency of hospital admissions (RR = 0.78 95% CI 0.46–1.30) nor the number of visits to primary care providers (RR = 0.83, 95% CI 0.63–1.09) differed by allocation group at 12-months post-randomization. Compared to TAU, CATCH-ED participants saw a reduction in disease-specific quality of life (QoLI20 total score) (-9.12, 95% CI -17.57 to 4.31), however, this instrument had a substantial number of missing data (only 50% of participants had total scores at both time points) and should be considered cautiously. Due to the extent of missing item-level data, the analysis was repeated with the global indicator (“How do you feel about your life as a whole?”), which has a reliability coefficient α >0.90 with the 20-item scale [31]. This global indicator did not show any statically significant differences between groups at 12 months (-0.19, 95% CI -0.36–0.74), adjusted for baseline values.

## Discussion

Our study found that after 12 months, frequent ED users with mental health conditions who received a brief intensive case management intervention had a 14% decrease in ED visits compared to those who received usual care, however, this decrease was not statistically significant. We also did not observe statistically significant differences between intervention and usual care participant groups for any other service use or health related secondary or exploratory outcome. The finding of a reduction in disease specific (mental health) quality of life, which decreased in the intervention compared to usual care group, should be interpreted with caution given the extent of missing data for this outcome.

Our findings are supported by a recent systematic review and meta-analysis, noting that care coordination strategies, including case management, are not effective in reducing hospital use among adults with mental illness [17]. Nonetheless, this study adds to the mixed but growing literature regarding the effectiveness of case management in reducing ED visits among frequent ED users. Among studies with randomized designs, two have shown that case management can lead to significant reductions in ED use among frequent ED users [15, 16], one saw increased ED use in the intervention group [32], while a recent trial showed no statistical difference between groups [33]. The high heterogeneity between studies, most importantly in how frequent users are defined, but also in participant demographic and clinical characteristics, the nature of the intervention (type and quality of case management) and system level-factors such as availability and accessibility of usual care services, may contribute to the lack of consistent findings and certainly limits the ability to compare findings between studies. Similar to our study, the most recent trial used the same definition of frequent users (≥5 ED visits in past 12 months), was held in a country with universal health insurance coverage and privately delivered health care (Switzerland) and showed a trend towards reduced ED use which did not reach statistical significance [33]. In comparison, of the two case management trials which showed a statistically significant reduction in ED among frequent ED users, the first took place in the US [15], while the second, conducted in a country with universal health insurance (Sweden), used telephone-based case management, an arguably different intervention [16]. Finally, a prior negative trial in the US used care plans and identified frequent users as those ≥10 ED visits in a year [32]. None of these trials focused on frequent ED users experiencing mental illness or addictions. In addition to these challenges in the existing literature, it is worth noting that case management is a complex intervention, often poorly characterized in studies and lacks the well-defined fidelity measures of assertive community treatment [34]]; as a result, services received may vary greatly between individuals in the same study and certainly between studies and jurisdictions [34, 35], with services received by the control group approximating those received by the intervention group in some studies [35]. It remains unclear whether the failure to reduce ED visits in this population reflects the failure of intensive case management in principle or in actual delivery of the intervention.

The lack of consistent findings by this and earlier studies suggests the need to re-examine the use of targeted case management interventions for frequent ED-user populations. Firstly, not all frequent ED use may be preventable or inappropriate [2, 7], and frequent ED users with complex health, mental health and substance related conditions in particular, such as our participants, may have crisis needs that cannot be addressed by available community-based services [36, 37]. Qualitative findings from our study indicate that participants perceived the ED as the only available, accessible and acceptable option for crisis care [7]. Furthermore, CATCH-ED study participants reported that visits to the ED were often sanctioned or encouraged by community-based providers who were commonly not available to address urgent or emergent crisis needs [7]. It is important for future research to examine the service needs and preferences of people with mental illness and addictions and the availability of appropriate community based services, particularly when planning efforts to reduce frequent ED use [7, 36, 37]. The availability of alternative services for urgent mental health conditions, including crisis respite services and urgent care centres, may help reduce ED visits by providing services which are currently only accessible to patients who visit the ED. For example, recent research has found that peer respite programs can reduce the odds of use of inpatient or emergency services among adults receiving publicly funded behavioural health services in the United States [38]. Secondly, brief case management has been shown to be effective in reducing psychiatric rehospitalisation [39] and improving other outcomes for *homeless* adults when provided at the point of discharge from various institutions, perhaps because these participants were specifically lacking connections to community-based resources. In contrast, community-dwelling frequent-ED users who are already well-connected to health and social services, including primary care [2], may not benefit from additional community-based services. For example, a 3-month CTI program failed to reduce use of acute care use (emergency department visits or hospitalization) among housed community-dwelling veterans with serious mental illness discharged from hospital psychiatric inpatient units [40]. Roland and Abel (2012) have highlighted several misconceptions around reducing ED use among frequent users [41], questioning the introduction of new services, especially when, as in our study, such services cannot replace the availability, comprehensiveness, and accessibility of the ED. Finally, while not observed in the current study, others have noted that care coordination strategies appear to improve patient experiences and outcomes; McWilliams (2016) argues that better patient care is in itself a worthy cause, even in the absence of cost savings or reduced service utilization [42].

Our study is not without limitations. Firstly, due to the nature of the intervention, participants and research staff were not blinded to treatment allocation. Secondly, the intervention’s effectiveness was assessed at 12-months, which may not have been long enough for potential benefits to show group differences in this population. However, an earlier trial demonstrated cost-effectiveness of case management for frequent ED users at both 12- and 24-months [15].

In conclusion, we found that compared to usual care, a brief intensive case management intervention did not result in reduced ED use among a population of frequent ED users with mental illness or addictions in Toronto, Canada. Future studies need to examine the availability, accessibility and appropriateness of existing community based services for people with complex health, mental health or addiction needs.

## Supporting information

S1 File Study protocol. (DOC)

S1 Table Consort checklist. (DOC)
