# Supplementary material for: The effect of brief case management on emergency department use of frequent users in mental health: Findings of a randomized controlled trial
Source: PLoS One. 2017 Aug 3;12(8):e0182157. doi: 10.1371/journal.pone.0182157 (PMC5542632; doi:10.1371/journal.pone.0182157)
Supplement: S1 File — (DOC) [file pone.0182157.s001.doc]

**PROTOCOL**

**Coordinated Access to Care from Hospital Emergency Departments (CATCH-ED):**

**Assessing Effectiveness and Cost Effectiveness**

**Background and Rationale**

Frequent users of Emergency Departments, across several jurisdictions, share several challenges, including low socioeconomic status, homelessness, mental illness/ addictions, and multiple medical co-morbidities. This population uses a disproportionate amount of acute care resources. Within the Toronto Central Local Health Integration Network (TC-LHIN), approximately 2% of frequent users (>20 visits annually) account for 20% of the total ED visits by this group (TC-LHIN, 2012). The emerging profile of frequent users suggests that current health care resources are not sufficient to adequately support a population that is inherently challenging to engage (Althaus, 2011).

Emerging evidence suggests that brief case management interventions are effective in reducing the gap between institutional and community-based care and improving continuity of care (Susser et al, 1997; Dixon et al, 2009). When continuity of care and treatment gaps are addressed, individuals with serious mental illness and/or chronic physical conditions experience improved health outcomes that are also associated with reduced hospital and community costs (Adair et al, 2005; Mitton et al, 2005; Cabana & Jee, 2004; Saultz & Lochner, 2005; Gill & Mainous, 1998; Gill et al, 2000). Similarly, both experimental and non-experimental studies support the effectiveness of case management interventions in decreasing ED visits and hospitalizations for frequent users of EDs (Shumway et al, 2009, Althaus et al, 2011). In a large before and after program evaluation of case management interventions for frequent users of EDs in 6 counties in California, a decrease of 30% in ED visits and 17% in hospital costs was achieved one year after program enrolment. Findings in year 2 of the program demonstrated further improvements, with an estimated decrease in ED visits of 60%, inpatient days of 62% and hospital costs of 62% (Linkins et al, 2008). Experimental studies have provided further support for the effectiveness of case management interventions (Althaus, 2011). A randomized controlled trial of 252 frequent users by Shumway confirmed significant reductions in ED use and ED costs 12 months after enrolment in a case management program. The intervention also led to significant decreases in homelessness and alcohol use and improvements in accessing income support and health cards (Shumway, 2008). A recent systematic review by Althaus concluded that it may be beneficial to patients and economically justifiable for hospitals to implement case management for frequent users (Althaus, 2011).

**The Intervention**

The Toronto Mental Health and Addictions Acute Care Alliance (Alliance) is currently implementing a novel model of care for the frequent ED user population with mental health and/or addictions problems. The goal of the intervention is to support their transition to community-based resources and reduce avoidable ED visits and hospitalizations. Coordinated Access to Care from Hospital Emergency Departments (CATCH-ED) currently connects mental health and addictions frequent ED users with a community-based transitional mental health case manager in each of three Alliance Hospital EDs ( St. Michael’s, St. Joseph’s Health Center, the Center for Addiction and Mental Health). The transitional case managers have access to a range of community support options to facilitate the development of individualized, integrated responses to better meet the person’s mental health, addictions and physical health needs in the community and reduce their need to rely on EDs and hospital admissions for health care. Support options include peer support and access to primary and urgent psychiatric care, and the creation of a comprehensive interdisciplinary team for program recipients. The primary care services for this project will be accessed through Community Health Centres (CHCs) and, potentially, from Family Health Teams (FHTs).

This model draws from the experience of the Coordinated Access to Care for the Homeless (CATCH Homeless) program, a made-in-Toronto variant of a Critical Time Intervention (CTI) model that provides transitional support to homeless people with complex, unmet health care needs in the community. The adapted CTI model includes three phases. During Phase 1 (month 1) program goals include assessing participant needs, strengths and resources, providing practical needs assistance and developing individualized treatment and support plans; Phase 2 (months 2-3) is focused on ongoing assistance and on facilitating and testing connections/referrals to appropriate long term community based supports; in Phase 3 (months 3-4) care is transferred to the support network developed during phase 2. Program activities include home visits and assertive outreach, introduction of participants to new service providers and discussion of the comprehensive care plan with all members of the support network. Furthermore, transitional case managers offer crisis intervention, supportive therapy, and assistance in obtaining income support and housing. CATCH-ED will serve to expand CATCH to increase the range of populations served; the range of services offered (e.g., team-based primary care in CHC and FHT settings, trauma-informed group services, and solution-focused therapy/support provided in non-ED settings); and possibly, the range of technologies used to connect and keep people connected with care providers (e.g., home/community-based monitoring of co-morbid medical conditions; use of portable telemedicine equipment to support nurses in the community; and telemedicine-facilitated consultation with psychiatrists). The CATCH-ED program model provides transitional case management support over 3-4 months, offering timely, potentially cost-effective access to needed services in a coordinated way.

**Implementation**

In February, 2012, the Alliance, a collaborative of 7 TC LHIN teaching hospitals, implemented CATCH-ED at 3 TC-LHIN Hospitals, in partnership with 4 CHCs and 6 Community Mental Health Agencies, targeting frequent ED users (>5 visits in the previous 12 months). An implementation evaluation is currently underway to assess challenges and facilitators of implementation at each site, fidelity of the intervention to the program model and stakeholder and consumer perspectives on the intervention for the purposes of further program development / adaptation. Implementation evaluation will be followed by an outcome evaluation, using a randomized controlled design. CATCH-ED is currently supported by the Alliance, the TC-LHIN, the Ontario MOHLTC and BRIDGES (Building Bridges to Integrated Care, Department of Family and Community Medicine, University of Toronto). The Alliance facilitates program partner meetings and implementation of the intervention. The TC-LHIN has provided funding to the CHCs to increase capacity for frequent users and trauma-informed services / counseling. A senior TC-LHIN manager participates in the Frequent Users Advisory Committee chaired by the Alliance. BRIDGES is providing funding for the implementation and outcome evaluation, with an anticipated time of completion of March 31st, 2014. BRIDGES, in addition, provides infrastructure and support for data collection and a cost-effectiveness evaluation of the program through the Applied Health Research Centre (AHRC) at the Li Ka Shing Knowledge Institute, St. Michael’s Hospital. The Ontario MOHLTC has provided start up funding for the implementation evaluation and will be participating in further discussion on expansion of the program to other sites in Ontario.

The project team, led by the Alliance, is based at St. Michael’s Hospital. Additional project partners include 6 community mental health agencies (COTA Health, TNSS, PARC, Reconnect, CRCT, Sound Times), and 4 CHCs (Regent Park, Riverdale, Parkdale and Queen West CHC). The project team is supported by the Mental Health and Addictions Frequent Users Advisory Committee (FUAC, comprised of frontline clinicians, people with lived experience of mental health and addictions problems, other service providers and decision-making stakeholders). FUAC will review challenges in implementation, review and finalize interview questionnaires for the implementation and outcome evaluation, interpret evaluation findings, guide program adaptations, provide feedback on the policy and clinical relevance of results, and participate in knowledge exchange events at the evaluation study end. The committee has facilitated the recruitment of project partners, and implementation of the program model at St. Michael’s Hospital, the Centre for Addiction and Mental Health and St. Joseph’s Health Centre.

**Research Team**

The research team is led by Dr. Stergiopoulos and includes: Dr. Tim Guimond, Dr. Stephen Hwang, Dr. Mel Kahan, Dr. Paul Kurdyak, Dr. Pat O’Campo, Dr. Howard Ovens and Dr. Don Wasylenki. The research team has considerable experience in program implementation, research methods, biostatistics, as well as policy relevant research related to ED use and vulnerable populations. Prior interventions’ research experience by team members includes the development and implementation of CATCH Homeless and the implementation and outcome evaluation of the At Home / Chez Soi Research Demonstration Project, a randomized controlled trial of Housing First in the Canadian context, delivering rent supplements as well as intensive case management and assertive community treatment for homeless people with mental illness.

**Study Objectives and Hypotheses**

This 2-year randomized controlled trial proposes to use multiple, longitudinal quantitative measures to assess the impact of CATCH ED on health outcomes and acute care service utilization for frequent users of ED (>5 visits/yr) 12 months after program enrolment. By better coordinating health and social services and by making diverse services more readily available in the community, CATCH-ED will offer frequent users a full array of services and good continuity of care. We hypothesize that continuity of community-based care will be associated with better health outcomes and decreased reliance on Emergency Department use and days in hospital during the 12-month follow up period.

The primary research question is: Is a brief case management intervention program (CATCH-ED) effective in reducing ED visits for frequent users of EDs (>5 ED visits in the past 12 months)? The primary measure of effectiveness is the reduction in ED use, 12 months after program enrolment.

The secondary research question is: Is the intervention effective in improving health outcomes for frequent users of EDs (>5 ED visits in the past 12 months)? The secondary measures of effectiveness are changes in: 1) inpatient care utilization; 2) health status; 3) mental health; 5) substance use; 6) quality of life and 7) service satisfaction, 12 months after program enrolment.

Furthermore, an economic evaluation will be completed by the AHRC. The objective of the economic evaluation is to compare the cost-effectiveness of the CATCH-ED intervention with usual care, using patient-level data from the randomized clinical trial and linked administrative data at the Institute for Clinical Evaluative Sciences (ICES), Toronto.

**Methods - Outcome Evaluation**

The study design is a randomized controlled trial in which multiple longitudinal outcome measures will be collected on both the intervention and control groups to assess client characteristics and selected outcomes of interest.

Frequent users will be recruited to the study over a 9-month period and followed for 12 months. Program participants will complete survey interviews at baseline, 6 and 12 months to assess health status, mental health, substance use, self reported acute care service use, service satisfaction and their working alliance with their primary clinician. Self-reported acute care use will be complemented by administrative data linkage to examine acute care service use 12 months prior and 12 months following referral to the intervention. Participants will also complete survey interviews at 3 and 9 months to assess their service use and to confirm their contact information and any time spent outside the Province during the previous three-month period.

*Sample Size Calculation*

In determining a sample size calculation, three important factors were considered: 1) participants are eligible by being at the extreme of the distribution of emergency visits in the overall population and hence regression to the mean may play an important role in any observed change over time, 2) accessing emergency services may in fact provide a treatment that may contribute to change over time in an 'untreated' group, 3) previous studies have found that emergency visits tend to follow a negative binomial distribution (a count distribution with highly right skewed data), and 4) a proportional reduction in the rate of emergency department visits is the desired primary measure of impact of this intervention. To account for these factors, published data of Shumway et. al. (2008) was used to estimate the distribution of emergency department (ED) visits in an untreated group and its natural reduction over time; their study of frequent ED users (>5 visits/year) included a ‘usual care’ group and reported the mean and standard deviation of ED visits over 2 years of follow-up in 6 month blocks. The sample size calculation was performed using the formula derived by Toshiro Tango (Statistics and Probability Letters 79 (2009) 466-472) which uses a Poisson type outcome and assumes a random effect for each individual, which can accommodate a negative binomial or other more dispersed distribution. This approach also takes into account any fixed effects that covariates may have in both the pre and post-intervention period. Ultimately, this sample size calculation proposes that a sample size of 64 in each arm would allow the detection of a 20% reduction in ED visit rates (the minimum clinically significant difference we would like to be able to demonstrate). Since up to 30% attrition is expected, among participants, the final sample size will be 166 participants, 83 in each arm.

*Randomization process*

Participants will be recruited among successive referrals from participating TC-LHIN Hospitals. Participants able to provide capable informed consent will be invited to participate to the study. A research coordinator or research assistant will obtain written informed consent and complete a baseline interview prior to randomization to CATCH-ED vs. treatment as usual. Randomization will be allocated through the web-based electronic case report forms (eCRFs.) The randomization sequence will be generated by random computerized sequence in variable block sizes.

*Longitudinal Follow -Up*

Study participants will be interviewed at baseline, 3, 6, 9 and 12 months following program enrolment. The study protocol requires face-to-face interviews at each of the five time points. Study participants will receive a payment of $50 for the baseline, 6 and 12 month interviews (duration of one hour) and $25 for the 3 month and 9 month follow up interviews (duration of 15 mintues). Study participants will be followed intensely by a research assistant, especially trained in locating and contacting individuals with mental health and addictions problems. Baseline interviews will be completed within two weeks of program referral. There will be a four-week window for data collection at the 3-month, 6-month, 9-month and 12-month time points. Interviews will be conducted between two weeks prior to and after the scheduled follow up date. Data will be considered missing for the particular time point if the participant is not interviewed within the interview windows. Research assistants will contact participants each month to confirm contact information. Additional strategies to minimize study attrition will include collecting names and phone numbers of family ,and /or acquaintances and /or service providers (with participant consent) to help maintain participant contact.

**Data Collection Plan and Data Management**

The Data Coordination Centre (DCC) will be the Applied Health Research Centre (AHRC), an academic research organization based at St Michael's Hospital. AHRC will use software called Medidata RAVE ([www.mdsol.com](http://www.mdsol.com/)) to create the web-based electronic CRF (eCRF). All study data will be securely stored on local servers at St Michael’s Hospital throughout the duration of the study and for up to 10 years after the study is complete. All study subjects will be identified in the database by a unique study ID number. Linkages between the patient name/contact information and the study ID will be retained at the local site and not shared with the study DCC or outside the institution. At the end of the study (after all analyses are complete) the DCC will transfer all study datasets over to the study PI. Data will only be accessible by authorized study site personnel and authorized central DCC personnel. Authorized personnel receive a username and password which is unique, and database access is controlled by the DCC in collaboration with the Principal Investigator.

Three members of this research team (VS, SH, PO) are Toronto site principal investigators of the At Home / Chez Soi Research Demonstration Project on Homelessness and Mental Health, a large longitudinal national program evaluation funded by the Mental Health Commission of Canada. This proposed study will utilize data collection instruments and knowledge gained from the At Home / Chez Soi study to help engage and follow frequent users of the ED. Survey interviews will be completed by research assistants (RA). The RAs, supported by a Research Coordinator (RC), will be responsible for recruiting study participants, collecting data from CATCH-ED participant clinical records to ascertain clinical diagnoses and frequency of contact with CATCH ED clinicians and administering participant survey interviews at entry point into the program and at 3, 6, 9 and 12 months following enrolment. RAs will be updating the tracking information at all participant interviews and at monthly intervals through telephone follow up with each participant. A brief summary of client measures is provided below.

*Socio-demographic variables*: Research staff will collect the following information: basic demographic information, residential status, income support status.

*Health Status*: Specific health status measures will include the SF-12, the EQ-5D, the Colorado Symptom Index, the Quality of Life Index (QoLi-20) and the alcohol and drug module of the Addiction Severity Index.

*SF- 12:* This 12-item self-report measure of generic health status is a shorter version of the SF-36 Health Survey designed to reproduce the Physical Component Summary (PCS) and Mental Component Summary (MCS) scores. It has an administration time of 2 minutes. There are 2 questions concerning physical functioning, 2 questions on role limitations because of physical health problems, 1 question on bodily pain, 1 question on general health perceptions, 1 question on vitality, 1 question on social functioning, 2 questions on role limitation because of emotional problems, and 2 questions on general mental health (psychological distress and psychological well-being). It has excellent psychometric properties. (Ware et al, 1996).

*EQ-5D:* EQ-5D is a self-administered standardized measure of health status developed by the EuroQoL Group in order to provide a simple, generic measure of health for clinical and economic appraisal. It provides a simple descriptive profile and single index value for health status. The EQ-5D descriptive system has 5 dimensions: mobility, self-care, usual activities, pain/discomfort and anxiety/depression. Each dimension has 3 levels: no problems, some problems and severe problems. The visual analog scale records the respondent’s self-rated health on a vertical, visual analogue scale where endpoints are labeled “best imaginable health state” and “worst imaginable health state”. This information can be used as a quantitative measure of health outcome as judged by the individual respondents (The EuroQuol Group, 1990; Brooks, 1996).

*Quality of Life Index (QoLI-20):* The original scale was designed to assess the quality of life of people with severe and persistent mental illness. It is a structured self-report interview, conducted by a trained non-clinical interviewer, and elicits participants’ ratings of their quality of life. There are 7 subjective scales (living situation, everyday activities, family, social relationships, finances, safety, and satisfaction with life in general) and 4 objective scales (everyday activities, enough money, family contacts, and contacts with friends). The 20-item version was developed by Uttaro et al. (1999) using item-response theory and it has retained excellent psychometric properties.

*Colorado Symptom Index (CSI):* The CSI was designed specifically for individuals with mental health problems. It is a 14-item instrument which assesses the presence and frequency of symptoms of mental illness experienced within the past month. Responses are provided using a 5-point Likert scale with answer choices ranging from 0 (not at all) to 4 (at least every day). A higher score indicates a higher level of symptoms. The Colorado Symptom Index is a widely used tool in research as a self-report measure of psychiatric symptomatology. The CSI has been reported to have excellent internal consistency (.92) and test-retest reliability (.71). Evidence of the CSI’s validity is strong as CSI scores have been proven to distinguish between individuals with and without mental health service needs and were significantly correlated with functioning(Boothroyd and Chen, 2008).

*ASI:* The alcohol and drug module of the Addiction Severity Index (ASI), the most widely used instrument in the field, will be utilized. The ASI is a structured interview instrument that yields information about the severity of lifetime and recent (previous 30 days) drug and alcohol use (McLellan et al, 1980). Five indices will be used to measure substance abuse-the number of days that alcohol or substances were abused in the past month, the amount of money spent on alcohol or drugs in the past month, and the participants’ rating of the need for alcohol or drug use treatment. The ASI has good inter-rater and test-retest reliability and good concurrent and discriminant validity. It has been used extensively with the homeless population (Hwang et al, 2005).

*Participant Service Use:* Service use events will be tracked using both self-reported and administrative data. We will establish the number of hospitalizations, days in hospital and emergency department visits during the year prior to program enrolment as well as the 12 month follow-up period. We will also collect data on non-acute service use, such as mental health, substance use, and primary care visits, using service use forms adapted from the At Home / Chez Soi project on homelessness and mental health.

*Service satisfaction – SSS10:* This scale is a shorter version of the SSS-30 (Greenfield & Attkisson, 2004) which was extensively tested in Alberta and in a few services in British Columbia (Adair et al, 2005). Participants will be asked whether they had received any assistance with an array of different care needs. The three needs categories will include: coordination of services; family and social networks and community linkages; and life skills training, support and practical needs assistance.

*Working Alliance Inventory-Participant (WAI-PAR):* This 12-item self-report measure (modified from the original 36 item version) is designed to assess the working-alliance construct based on the theory that a working alliance common to all therapeutic relationships grows out of client-therapist agreement on therapy goals, agreement on therapy tasks, and development of a strong relational bond between client and therapist (Busseri et al, 2003). There are 4 items (chosen from the longer scale using factor analysis) in each of three subscales: task, goal and bond. Items are rated on 7-point scales (1=never, 7=always). Ratings yield task, goal and bond subscale scores as well as an average score based on all items. The scale has good psychometric properties, with mean reliability estimates ranging from .79 to .97, with a modal estimate of .92 (Horvath & Greenberge, 1989; Hanson et al, 2002).

*Administrative Data Linkage*

Data linkage will be conducted at the Institute for Clinical Evaluative Sciences (ICES) where population based health information is available, at the patient level, for all Ontarians using formal health services. Subjects will be linked deterministically using their Ontario Health Card Number (encrypted at ICES to preserve anonymity). Health administrative data will be used to identify individuals’ use of hospital and physician services in a 12-month observation window preceding and following their enrolment to the CATCH-ED program. Health service use will be examined by the National Ambulatory Reporting System (NACRS), the Discharge Abstract Database (DAD), the Ontario Mental Health Reporting System (OMHRS) and the Ontario Health Insurance plan (OHIP) for ED visits, outpatient clinics and inpatient hospitalizations.

*Other Administrative Data*

Provincial databases, including the Ontario Drug Profile Viewer and the Office of the Registrar General, will be consulted in the case where participants are lost to follow up at 12 months in order to ensure that health service utilization is not affected by death or relocation to another province. Informed consent to consult these databases will be collected from participants at study entry.

**Analysis Plan**

We will calculate descriptive statistics (mean, standard deviation, quartiles) and construct graphs (histograms, scatter plots, box-plots) to characterize the clients at baseline with respect to socio-demographic factors, housing status, health status, and health service use. Baseline group comparisons will be conducted using chi-square test or Fisher’s exact test (for categorical variables), t-test or Wilcoxon rank-sum test (for continuous variables). We will construct plots to describe longitudinal changes in primary and secondary outcomes over time. All analyses will use an intent-to-treat perspective and will be compared with a per-protocol analysis. The primary outcome evaluates the proportional change in rate of emergency department visits (ED visits) between pre-intervention and 6-month time intervals post-intervention; some secondary outcomes (number of hospitalizations, days in hospital) are also count measures, negative binomial outcomes will be compared with Poisson regression accounting for overdispersion to determine the overall treatment effect. While it is usually customary to match the sample size calculation technique to analysis approach, in this case insufficient published data is available to calculate a sample size for a marginal techniques (Generalized Estimating Equations - GEE) that is proposed as our primary analytic technique. While GEE models represent the main question being studied (the impact of the intervention on population measures of ED use), conditional models can also provide useful information and sample size techniques require fewer assumptions. For this reason, both marginal and conditional (random effects models) will be fit and compared during the data analysis phase of this study. The choice of the most appropriate representation of the overdispersion of data (Negative binomial versus the use of an overdispersion factor in a poisson regression) is highly dependent on the variance structure observed in the collected data (Ver Hoef & Boveng, 2007); hence, the covariance estimates of the observed data will inform final model selection. GEE and mixed effects models will be compared to account for the repeated measurement of participants. A literature review and the presence of clinically significant differences in baseline data will determine which, if any, covariates require adjustment in the final model, and these will be included as covariates in the generalized linear regression models. Other secondary normally distributed outcomes (quality of life, service satisfaction) with be analysed using linear regression using the same principles of adjustment for clinically important covariates, and marginal versus conditional approaches.

Given the possibility of missing data in following participants for 12 months who may have unstable housing or community connections, imputation methods will be used to estimate the overall impact of missing data on outcomes. Administrative data from the Institute of Clinical and Evaluative will be used for the purposes of evaluating the primary outcome, and comparing the impact of missing data on outcomes.

*Economic Evaluation Plan*

The objective of the economic evaluation is to compare the cost-effectiveness of the CATCH-ED intervention with usual care, using patient-level data from the randomized clinical trial and linked administrative data at the Institute for Clinical Evaluative Sciences (ICES), Toronto. The Ontario MOHLTC perspective will be used in the analysis. In Ontario, the Ministry pays for physician visits, laboratory tests, imaging, hospitalization, emergency department visits, same day surgery/ procedure, complex continuing care and some home care services. In addition, the Ministry pays for prescription drugs for those older than 65 years old, live in a Long-Term Care Home or a Home for Special Care or enrolled in special programs such as the Ontario Disability Support Plan (ODSP). The time horizon for the economic evaluation will be 12 months (maximum follow-up of the trial).

The primary outcome will be the incremental cost per quality-adjusted life-year (QALY) gained though the intervention compared with usual care. The secondary outcomes include the incremental cost per ED visit avoided and incremental cost per hospital-day avoided.

Measuring QALYs: A QALY is a year of life experienced with a particular health-related quality of life (HRQoL) or health state utility, as represented on a scale from 0 to 1, where 0=death and 1=full health. The EQ-5D and SF-6D (converted from SF-12), generic preference-based instruments that have been used in mental health patients, will be used to assess patients’ health state utility at baseline, 6 months and 1 year. We will calculate each patient’s QALY from the utility scores using the area-under-the-curve approach, with linear interpolation between assessment points (Figure 1).


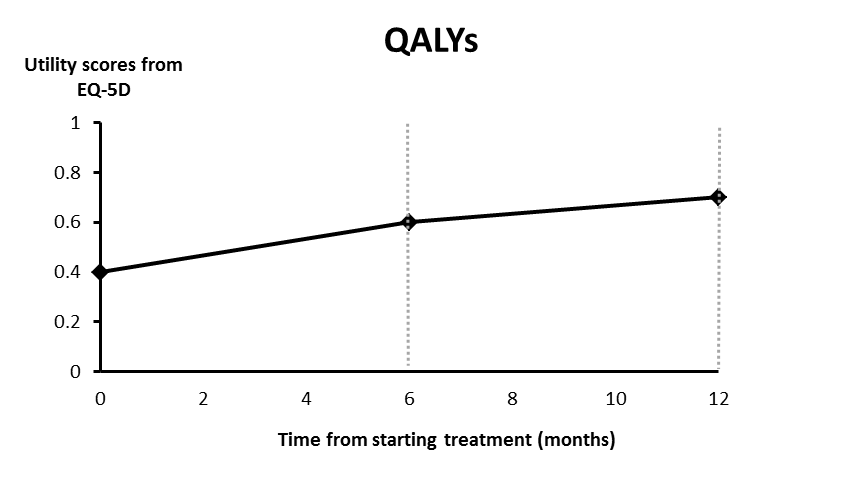


QALY0-90 days

QALY6 -12 months

QALY0-6 months

Figure 1. Calculating QALYs from EQ-5D utility scores

Measuring resource use and costs: We will obtain the health care costs of each patient from linked administrative data at ICES (Table 1). Informed consent will be collected from each participant for linking study data to the administrative data at ICES. The two dataset will be linked using patients’ OHIP numbers.

Table 1. Data source

| **Resources** | **Sources** | **Variables** |
| --- | --- | --- |
| Hospitalization | Discharge Abstract Database | Cost estimated using Resource Intensity Weight (RIW) and cost per weighted case |
| Outpatient laboratory / imaging services and physician services (including inpatient, office and undefined service) | Ontario Health Insurance Plan (OHIP) Claims Database | OHIP billing cost estimated using utilization frequency of each service and cost per visit from OHIP fee schedule |
| Emergency Room visits / Same Day Surgery or procedure | National Ambulatory Care Reporting System (NACRS) and Same Day Surgery | Cost estimated using RIW and cost per weighted case |
| Outpatient prescription drugs | Ontario Drug Benefit Plan (ODB) | Drug cost and dispensing fee |
| Home care services | Home Care Database | Utilization frequency for each type of home care service |
| Complex continuing care | Continuing Care Reporting System | Case-mix index and duration between events or RWPD |

RWPD: RUG Weighted Patient Day is calculated by multiplying the case-mix index (CMI) and number of days for a particular RUG event. It accounts for admissions, assessments, re-entries, discharges, etc.

Calculating incremental cost per QALY gained: We will estimate average and incremental costs and QALYs for each group using multiple regressions:


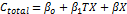


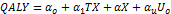


where x is a vector of patient characteristics (e.g., age, sex), U­0 is the utility at baseline,10 TX­ is a treatment variable (TX=1 for intervention group), and the regression estimates β1 is the incremental cost and α1 is the incremental QALY.

Calculating incremental cost per ED visit avoided: We will estimate average and incremental costs (excluding ED visit costs) for each group using multiple regressions. The incremental cost per ED visit avoided is the incremental cost divided by the incremental number of ED presentations (more details in the statistical analysis section).

Calculating incremental cost per hospital-day avoided: We will estimate average and incremental costs (excluding hospitalization costs) for each group using multiple regressions. The incremental cost per hospital-day avoided is the incremental cost divided by the average number of hospital-days (more details in the statistical analysis section).

Calculating net benefit regression: A treatment is cost-effective if the ICER is less than what the decision maker is willing to pay (λ) for the extra benefit (QALY gained, ED visit avoided or hospital-day avoided):

∆C/∆E <λ

We can also express this decision in terms of incremental net benefit, ∆NB:

∆NB = λ ∆E - ∆C

A treatment is cost-effective if it has a positive ∆NB, the value of the intervention’s extra benefits outweighs its extra costs. It is easier to estimate confidence interval for ∆NB because it is not a ratio. We will estimate the incremental net benefit of the intervention (β1) using multiple regressions:

NB= β0 + β1TX + β**X**

We could identify subgroups for whom the intervention is especially cost-effective using interaction terms. We will conduct net benefit regression analysis using different values of λ and will represent uncertainty using cost-effectiveness acceptability curves.

**Potential Impact:**

In Ontario, access, coordination and timeliness of appropriate services have been identified as health system goals (Ministry of Health and Long Term Care, 1999). Frequent users of EDs, poorly connected to community-based services, are at high degree of repeated ED visits and poor health outcomes. Given the potential for adverse consequences and associated health care costs, it is important for health treatment systems and providers to develop and implement strategies to increase community-based treatment engagement for this group and contribute to health system effectiveness and cost-effectiveness. Grounded on evidence-supported clinical interventions, CATCH-ED has the potential to substantially decrease ED visits, health care costs, as well as improve the patient experience, communication across providers and sectors, and health and social outcomes for some of the most vulnerable people in our community. The model promotes integration of hospital, primary care and community mental health services and can be implemented in a variety of settings across the province, including medium and smaller size cities, with little new investment. It is aligned with a number of Ontario MOHLTC priorities, including the ED Wait Time Strategy, and the Access to Family Health Care for All, as it aims to reduce ED wait times by reducing ED visits and readmissions to hospital as well as connect high risk clients to primary care physicians and specialists and improve care transitions across sectors and between health providers.
